# Vitamin B6, Inflammation, and Cardiovascular Outcome in a Population-Based Cohort: The Prevention of Renal and Vascular End-Stage Disease (PREVEND) Study

**DOI:** 10.3390/nu12092711

**Published:** 2020-09-04

**Authors:** Isidor Minović, Lyanne M. Kieneker, Ron T. Gansevoort, Manfred Eggersdorfer, Daan J. Touw, Albert-Jan Voerman, Margery A. Connelly, Rudolf A. de Boer, Eelko Hak, Jens Bos, Robin P. F. Dullaart, Ido P. Kema, Stephan J. L. Bakker

**Affiliations:** 1 Department of Laboratory Medicine, University of Groningen, University Medical Center Groningen, 9700RB Groningen, The Netherlands; i.p.kema@umcg.nl; 2 Department of Internal Medicine, University of Groningen, University Medical Center Groningen, 9700RB Groningen, The Netherlands; l.m.kieneker@umcg.nl (L.M.K.); r.t.gansevoort@umcg.nl (R.T.G.); dull.fam@12move.nl (R.P.F.D.); s.j.l.bakker@umcg.nl (S.J.L.B.); 3 DSM Nutritional Products, CH-4303 Kaiseraugst, Switzerland; manfred.eggersdorfer@dsm.com; 4 Department of Clinical Pharmacy and Pharmacology, University of Groningen, University Medical Center Groningen, 9700RB Groningen, The Netherlands; d.j.touw@umcg.nl (D.J.T.); a.j.voerman01@umcg.nl (A.-J.V.); 5 Laboratory Corporation of America® Holdings (LabCorp), Morrisville, NC 27560, USA; connem5@labcorp.com; 6 Department of Cardiology, University of Groningen, University Medical Center Groningen, 9700RB Groningen, The Netherlands; r.a.de.boer@umcg.nl; 7 Unit of Pharmacotherapy, Epidemiology and Economics, University of Groningen, 9712CP Groningen, The Netherlands; e.hak@rug.nl (E.H.); h.j.bos@rug.nl (J.B.)

**Keywords:** vitamin B6, pyridoxal 5’-phosphate, inflammation, GlycA, cardiovascular

## Abstract

Background: a large number of studies have linked vitamin B6 to inflammation and cardiovascular disease in the general population. However, it remains uncertain whether vitamin B6 is associated with cardiovascular outcome independent of inflammation. Methods: we measured plasma pyridoxal 5’-phosphate (PLP), as an indicator of vitamin B6 status, at baseline in a population-based prospective cohort of 6249 participants of the Prevention of Renal and Vascular End-stage Disease (PREVEND) study who were free of cardiovascular disease. As indicators of low-grade systemic inflammation, we measured high-sensitivity C-reactive protein and GlycA; Results: median plasma PLP was 37.2 (interquartile range, 25.1–57.0) nmol/L. During median follow-up for 8.3 (interquartile range, 7.8–8.9) years, 409 non-fatal and fatal cardiovascular events (composite outcome) occurred. In the overall cohort, log transformed plasma PLP was associated with the composite outcome, independent of adjustment for age, sex, smoking, alcohol consumption, body mass index (BMI), estimated glomerular filtration rate (eGFR), total cholesterol:high-density lipoprotein (HDL)-cholesterol ratio, and blood pressure (adjusted hazard ratio per increment of log plasma PLP, 0.66; 95% confidence interval (CI), 0.47–0.93). However, adjustment for high-sensitivity C-reactive protein and GlycA increased the hazard ratio by 9% and 12% respectively, to non-significant hazard ratios of 0.72 (95% confidence interval, 0.51–1.01) and 0.74 (95% confidence interval, 0.53–1.05). The association of plasma PLP with cardiovascular risk was modified by gender (adjusted P_interaction_ = 0.04). When stratified according to gender, in women the prospective association with cardiovascular outcome was independent of age, smoking, alcohol consumption, high-sensitivity C-reactive protein, and GlycA (adjusted hazard ratio, 0.50, 95% confidence interval, 0.27–0.94), while it was not in men (adjusted hazard, 0.99, 95% confidence interval, 0.65–1.51). Conclusions: in this population-based cohort, plasma PLP was associated with cardiovascular outcome, but this association was confounded by traditional risk factors and parameters of inflammation. Notably, the association of low plasma PLP with high risk of adverse cardiovascular outcome was modified by gender, with a stronger and independent association in women.

## 1. Introduction

Cardiovascular (CV) diseases are the leading cause of death globally [1]. Moreover, the societal burden of CV diseases will likely continue to rise due to aging, lifestyle factors and, paradoxically, better treatment of subclinical CV disease [2]. This underscores the need for non-conventional modifiable factors to complement and improve existing CV risk reduction strategies in the general population.

In light of this, vitamin B6 deficiency has gained considerable attention as a potential risk factor for CV disease [3,4,5,6]. Vitamin B6 is an essential micronutrient involved in >160 different biochemical processes that affect metabolism of amino acids, lipids, and neurotransmitters [7]. In the circulation, vitamin B6 exists predominantly as pyridoxal 5’-phopshate (PLP), which is used clinically to diagnose vitamin B6 deficiency [8].

An independent link between circulating PLP and CV outcome has been debated for decades and yet available evidence remains contradictory. While some studies have indeed suggested that this relation is independent [6,9,10,11], others have conjectured that the association between low circulating PLP and CV risk could be largely explained by inflammation [5,12].

Studies that have considered inflammation in the cardiovascular interpretation of vitamin B6, have based their assessment of inflammation mostly on the traditional early acute phase marker high-sensitivity C-reactive protein (hs-CRP). In view of the need for a comprehensive biomarker of the inflammatory response, proton nuclear magnetic resonance (NMR) spectroscopy has recently identified a novel biomarker, GlycA, that consists of the combined NMR signal from N-acetyl methyl moieties of the late acute phase proteins α1-antichymyotrypsin, α1-acid glycoprotein, haptoglobin, α1-antitrypsin, and transferrin [13,14]. The added value of GlycA over hs-CRP in the prediction of CV disease has been established by numerous studies, in which GlycA was associated with incident CV events independent of hs-CRP [15,16,17,18,19,20,21].

Hence, we aimed to investigate whether vitamin B6 deficiency is an independent risk factor for CV outcome in a population-based cohort, with specific consideration of the potential involvement of inflammation. To this end, we measured plasma PLP, hs-CRP, and GlycA in a large, extensively characterized, prospective population-based cohort. This cohort is part of the population-based Prevention of Renal and Vascular End-stage Disease (PREVEND) study [22].

## 2. Materials and Methods

### 2.1. Study Population

This study was conducted within the framework of the Prevention of Renal and Vascular End-stage Disease (PREVEND) study, an observational prospective cohort study which was set up to investigate the predictive value of urinary albumin excretion in relation to renal disease and CV outcome in the general population. The study design and recruitment procedures have been described in detail previously [23]. Briefly, non-pregnant participants (aged 28–75 years) free of type 1 diabetes mellitus were selected from the population of the city of Groningen, the Netherlands, to create a study population with a large variety in age and albuminuria levels. Baseline measurements were performed in 8592 participants between 1997 and 1998. For the present analyses, we excluded participants with a history of CV disease, which was defined as having coronary heart disease or having experienced a cerebrovascular accident, to avoid potential bias attributed to reverse causation. Additionally, participants of whom no plasma was available for quantification of PLP were excluded from analyses. The final study population that was available for analysis consisted of 6249 individuals who were free of CV disease at baseline and had no missing value for the focal variable, i.e., plasma PLP. The PREVEND study was approved by the medical ethics committee of the University Medical Center Groningen and was conducted in accordance with the Declaration of Helsinki, ethical approval code: METC 96/01/022. All participants provided informed consent.

### 2.2. Data Collection, Laboratory Measurements, and Definitions

Study participants completed two visits to our outpatient clinic for assessment of baseline data and for delivering 24 h urine collections. During urine collection, the participants were asked to avoid heavy exercise as much as possible. Participants were also instructed to postpone the urine collection in case of urinary tract infection, menstruation, or fever. The urine collections were stored at −20 °C. Prior to their first visit, all participants completed a self-administered questionnaire regarding demographics, history of CV and renal disease, smoking habits, alcohol consumption, and medication. Answer options of alcohol consumption included the following: no/rarely, 1–4 drinks/month, 2–7 drinks/week, 1–3 drinks/day, and 4 or more drinks/day. Information on medication and use of vitamin supplements was combined with information from the IADB.nl database, which contains pharmacy-dispensing data from community pharmacies in the Netherlands [24]. After an overnight fasting period and 15 min of rest at the outpatient clinic, venous blood was obtained from the study participants between 8.00 and 10.00 AM and immediately centrifuged at 4 °C. Subsequently, routine clinical chemistry measurements were performed the same morning and plasma samples were stored in a continuously monitored −80 °C freezer for future analyses. PLP was measured in fasting plasma by means of a validated routine high-performance liquid chromatography assay (Waters Alliance) with fluorescence detection (Jasco FP-2020; Jasco, Tokyo, Japan) [25], with inter-assay coefficients of variation (CVs) <2.5%. Vitamin B6 sufficiency, insufficiency, and deficiency were defined in accordance with generally accepted cut-off values as plasma PLP concentrations >30 nmol/L, in the range of 20–30 nmol/L and <20 nmol/L, respectively [26]. Hs-CRP and the novel biomarker GlycA [13,27] were measured as indicators of systemic inflammation by means of nephelometry (CVs <5.7%) and nuclear magnetic resonance (CVs <2.3%), respectively. Biomarkers of tobacco use and alcohol consumption, urinary cotinine and ethylglucuronide excretion, respectively, were quantified with commercially available DRI^®^ enzyme immunoassays on an Architect C8000 platform (Abbott, The Netherlands). Both assays were validated according to European Medicines Agency guidelines [28] with overall CVs <15%. Glucose, lipids, creatinine, and cystatin C were measured in plasma using standard methods as described previously, all with CVs <10% [29,30,31]. Muscle mass, fruit intake, protein intake, and generalized endothelial dysfunction were estimated by excretions of creatinine, potassium, ureum, and albumin, respectively, which were analyzed with routine laboratory assays (all CVs <10%) and estimated as the mean of the two 24 h urine collections. Estimated glomerular filtration rate (eGFR), was calculated using the Chronic Kidney Disease Epidemiology Collaboration (CKD-EPI) combined creatinine-cystatin C equation [32].

All blood samples were handled systematically to minimize variation and ensure reproducibility. All assays were performed by qualified laboratory personnel using de-identified samples.

### 2.3. Follow-Up and Ascertainment of Cardiovascular (CV) Events

Participants were followed from inclusion in 1997 or 1998 to 2011 and follow-up time was summarized among the individuals with censored data [33]. Participants were censored if they had moved from Groningen or to an unknown destination, or if they died from a non-CV or unknown cause. All events were coded according to the International Classification of Diseases, Ninth Revision (ICD-9) and the classification of interventions. As primary CV outcome, we used the composite of CV disease and CV mortality. Secondary analyses were performed for CV disease and CV mortality separately. For this study, CV disease was diagnosed if a participant had experienced or had undergone one of the following events: acute myocardial infarction (ICD-9 code 410), acute and subacute ischemic heart disease (ICD-9411), subarachnoid hemorrhage (ICD-9 430), occlusion or stenosis of the precerebral (ICD-9433) or cerebral arteries (ICD-9 434), coronary artery bypass grafting or percutaneous transluminal coronary angioplasty, and other vascular interventions such as percutaneous transluminal angioplasty or bypass grafting of aorta and peripheral vessels.

### 2.4. Statistical Analysis

Continuous data from Gaussian distributions are reported as mean ± standard deviation. Data from skewed distributions are presented as median [interquartile range (IQR)] and were log transformed where appropriate. Discrete data are shown as number (%). Baseline characteristics of our study population are shown for the overall population and according to vitamin B6 status. Cross-sectional associations between plasma PLP and other baseline variables were assessed by means of linear regression analysis, in which adjustments were made for the potentially confounding variables age and sex. From the linear regression analyses, we reported standardized betas and corresponding P-values to indicate strength and statistical significance of the associations.

To study the potential impact of low plasma PLP concentration on CV outcome, we performed Cox proportional hazard regression analyses. Several subjects had missing values for ≥1 baseline variables [i.e., smoking, alcohol consumption, systolic and diastolic blood pressure (SBP and DBP), albumin excretion (all ≤1.0%), GlycA (2.4%), total cholesterol:high-density lipoprotein (HDL) cholesterol ratio (3.0%), eGFR (4.7%), hs-CRP (16.2%)]. Because exclusion of subjects with missing values could result in biased prospective results, multiple imputation (fully conditional specification according to the Markov Chain Monte Carlo method) was used to obtain 5 imputed data sets (34, 35) which were subjected to the Cox regression analyses. Rubin’s rules were followed to obtain pooled estimates of the regression coefficients and their standardized errors across the imputed data sets [34]. Associations between log transformed plasma PLP and CV outcome were adjusted for potential confounders, including age, sex, smoking, alcohol consumption, body mass index (BMI), eGFR, albumin excretion, total cholesterol:HDL cholesterol ratio, SBP, and DBP in a stepwise fashion to create a confounder-adjusted model [7]. The confounder-adjusted model was subsequently adjusted for hs-CRP and GlycA separately and simultaneously (fully adjusted model). Effects of adjustment for hs-CRP and GlycA were assessed by quantifying the relative change in the hazard ratio (HR) point estimate via the following formula [35]:

[(HR after adjustment–HR before adjustment)/(1–HR before adjustment)] × 100(1)

Proportionality of hazards was investigated by inspecting the Schoenfeld residuals. Furthermore, linearity of the continuous prospective associations was tested by comparing non-linear restricted cubic spline models with three knots, i.e., at the 25th, 50th, and 75th percentile of the plasma PLP distribution, with corresponding linear models using χ^2^ tests. In sensitivity analyses, we investigated the impact of multiple imputation, by performing Cox regression analyses on the original, non-imputed, dataset. Furthermore, to account for oversampling of subjects with higher albuminuria levels in our study population, we conducted additional sensitivity analyses in which we performed Cox regression analyses by the use of complex survey design analyses [36]. Finally, we conducted sensitivity analyses in which we excluded participants who were taking supplements containing vitamin B6, in the three-month period before they were included in the study.

Statistical analyses were all conducted using SPSS 22.0 software (SPSS Inc.), with the exception of linearity tests of the continuous prospective associations which were performed in R version 3.2.3 software (The R-Foundation for Statistical Computing). Because of the general low power for interaction tests, interaction terms were considered to be statistically significant at two-sided *P*_interaction_ values of <0.10, as recommended by Selvin [37] and by the Food and Drug Administration authorities [38]. Furthermore, due to the high likelihood of low numbers of events in strata, and consequent low statistical power for Cox regression analysis, adjustment of stratified hazard ratios and interaction terms was limited to the main confounders of age, sex, smoking, alcohol intake, and the inflammation indicators hs-CRP and GlycA, where appropriate. All other two-sided *P*-values <0.05 were considered statistically significant.

## 3. Results

Baseline data are presented for the overall study population and according to vitamin B6 status in Table 1. The median overall plasma PLP concentration was 37 (IQR, 25–57) nmol/L. Vitamin B6 insufficiency and deficiency were identified in 1261 (20.2%) and 902 (14.4%) of the study participants. Women had a significantly lower plasma PLP concentration, 36 (24–57) nmol/L, compared to men, 39 (27–57) nmol/L (*p* < 0.001). Accordingly, vitamin B6 deficiency was more prevalent among women (508, 16%), compared to men (394, 13%) (*p* = 0.003). Plasma PLP was positively associated with level of education, moderate physical activity, alcohol consumption, urinary excretion of ethylglucuronide, potassium, and urea, HDL cholesterol, and eGFR in univariate regression models (all P < 0.05). Inverse univariate associations were found for age, BMI, smoking, cotinine excretion, SBP, DBP, coffee consumption, hs-CRP, GlycA, diabetes, glucose, total cholesterol:HDL cholesterol ratio, cystatin C, albumin excretion, and use of antihypertensives, antidiabetics, and statins (all *P* < 0.05). Adjustment for age and sex did not appreciably affect these baseline associations. 

**Figure 1:**
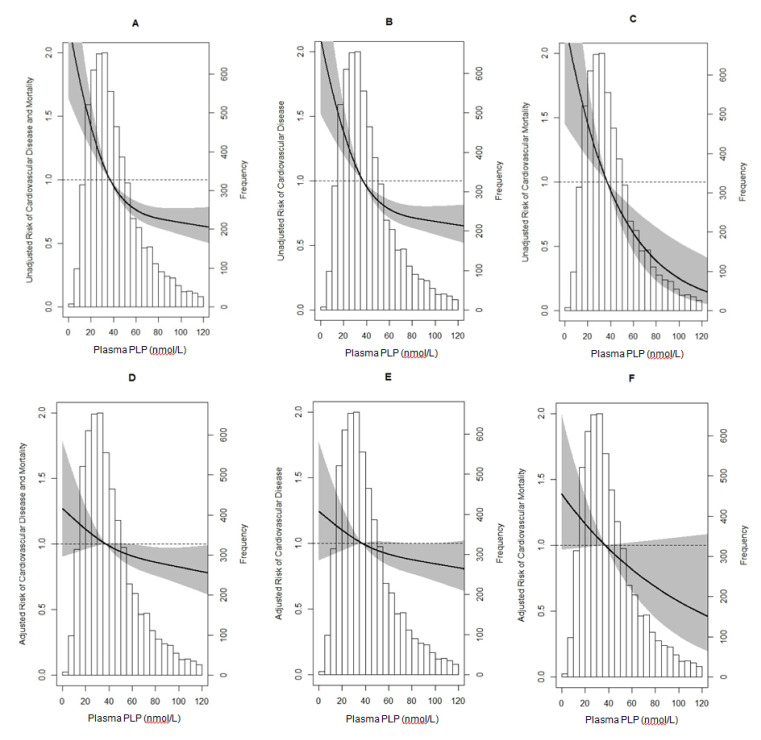
Unadjusted and fully adjusted continuous associations between plasma pyridoxal 5’-phosphate and cardiovascular outcome. Unadjusted associations for the composite cardiovascular outcome (figure **A**, *P*_nonlinearity_ < 0.001), cardiovascular disease (figure **B**, *P*_nonlinearity_ = 0.002), and cardiovascular mortality (figure **C**, *P*_nonlinearity_ = 0.10) were collectively adjusted for age, sex, smoking, alcohol consumption, BMI, eGFR, albumin excretion, total cholesterol:HDL-cholesterol ratio, systolic and diastolic blood pressure, hs-CRP, and GlycA (figures **D**–**F**, respectively). Abbreviations: hs-CRP, high-sensitivity C-reactive protein; BMI, body mass index; HDL, high-density lipoprotein; eGFR, estimated glomerular filtration rate; PLP, pyridoxal 5’-phosphate.

This study had a median follow-up of 8.3 years (IQR, 7.8–8.9 years) in which 409 incident non-fatal and fatal CV events occurred. In the same period, 379 participants developed CV disease and 77 died due to CV causes. The continuous term of log transformed plasma PLP was inversely associated with the composite CV outcome (HR 0.35; 95%CI, 0.25–0.49), Table 2. This prospective association was non-linear (*P*_nonlinearity_ < 0.001), Figure 1A. Adjustment for the potential confounders, age, sex, smoking, and alcohol consumption considerably attenuated the inverse association of plasma PLP with composite CV outcome (HR 0.60; 95%CI, 0.42–0.84). Additional adjustment for BMI, eGFR, albumin excretion, total cholesterol:HDL-cholesterol ratio, SBP, and DBP did not materially influence this association, resulting in a significant confounder-adjusted HR of 0.66 (95% CI, 0.47–0.93), Table 2. However, accounting for hs-CRP explained 9% of the association of plasma PLP with the composite CV outcome and increased the HR to a non-significant value of 0.72 (95% confidence interval (CI), 0.51–1.01). Of note, adjustment for GlycA had a slightly more pronounced effect, explaining 12% of the association and resulting in a non-significant HR point estimate of 0.74 (95% CI, 0.53–1.05), Table 3. The adjustments had similar effects on the associations with secondary end-points, i.e., CV disease and CV mortality, compared to the primary composite CV outcome, Table 2 and Table 3.

**Figure 2:**
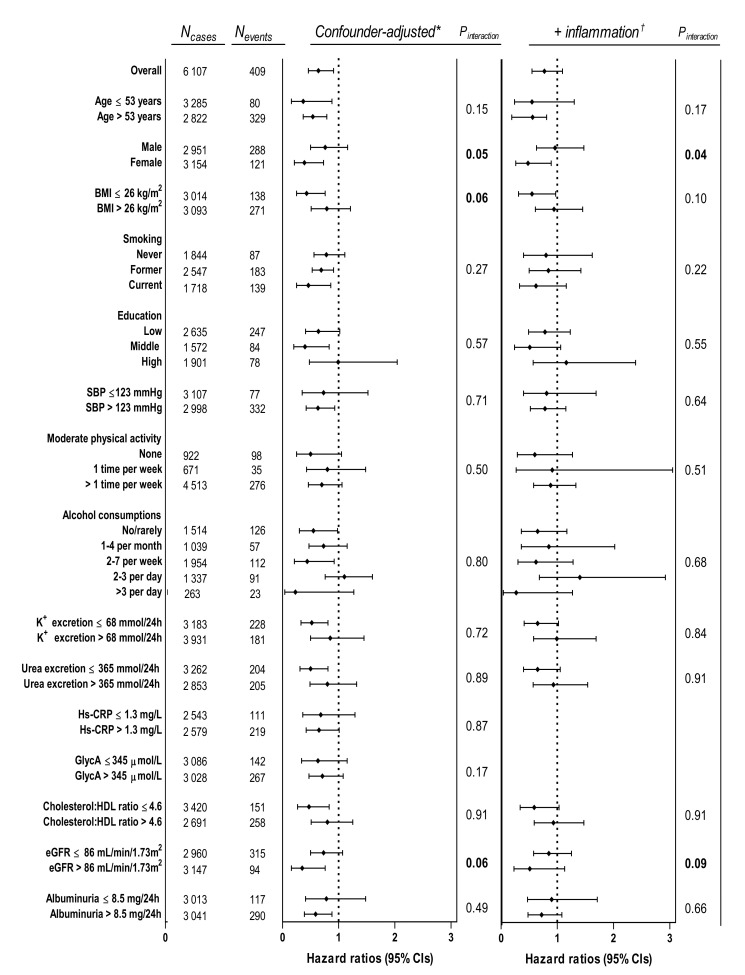
Stratified analyses for potential effect modification of the association between plasma pyridoxal 5’-phosphate and composite cardiovascular outcome. Hazard ratios indicate relative change in risk of composite cardiovascular outcome per increment of log transformed plasma PLP. * Adjusted for age, sex, smoking, and alcohol consumption. For strata according to one of these variables, adjustments were made for the remaining three covariates. ^†^ Additionally adjusted for hs-CRP and GlycA. Abbreviations: hs-CRP, high-sensitivity C-reactive protein; PLP, pyridoxal 5’-phosphate; BMI, body mass index; SBP, systolic blood pressure; HDL, high-density lipoprotein; eGFR, estimated glomerular filtration rate; CI, confidence interval.

In examining the association of plasma PLP with the composite CV outcome in subgroups of potential modifiers, we found that after adjustment for age, smoking, alcohol consumption, and inflammation the association was modified by sex (*P*_interaction_ = 0.04) and eGFR (*P*_interaction_ = 0.09), Figure 2. Low plasma PLP was independently associated with increased risk of the composite CV outcome in women (HR 0.50; 95% CI, 0.27–0.94), but not in men (HR 0.99; 95% CI, 0.64–1.51).

Point estimates from the sensitivity analyses on the non-imputed dataset, using complex survey design analyses, and after excluding participants taking vitamin B6-containing supplements, were not materially different from the reported data.

## 4. Discussion

In this large population-based cohort, we found no evidence for an independent relation between vitamin B6 deficiency, as assessed by plasma PLP concentration, and increased risk of CV outcome in the overall cohort. However, the association between plasma PLP and CV outcome was modified by sex and, to a lesser extent, eGFR. Notably, the inverse association between plasma PLP and CV risk was strong and independent among women, but not among men. 

Our data are in line with previous studies, reaffirming that plasma PLP is inversely associated with cardiovascular risk factors [12,39,40]. Accordingly, our study confirms that ageing and smoking could lower plasma PLP concentration, while moderate alcohol consumption may have an increasing effect [7]. The paradoxical effect of moderate alcohol consumption on plasma PLP concentration has been ascribed to the vitamin B6 content in beer [7]. However, a small randomized, diet-controlled trial has shown that, not only beer, but also post-meal consumption of red wine and spirits significantly increased plasma PLP concentration, compared to consumption of water [41]. These observations suggest that the beneficial effect of moderate alcohol consumption on plasma PLP may pertain to direct effects of ethanol on vitamin B6 handling by the human body. In addition, our data confirm the well-established strong inverse association between plasma PLP and inflammation [42]. While two small studies have suggested that this association could be a reflection of the effects of inflammation on plasma PLP concentration [43,44], causality of this relationship has not been formally investigated. Furthermore, our data on the difference in plasma PLP concentration between sexes correspond with previous observations that revealed lower concentrations in women than in men, and may be explained by a more pronounced age-related decline in plasma PLP concentration in women, compared to men [45]. However, processes responsible for this difference in plasma PLP concentration are yet to be identified. 

Several case-control studies, have argued in favor of an independent association between plasma PLP and various CV disease outcomes, both in gender-mixed populations [3,9,10,11], and in women [6]. Our data reveal that the association of low plasma PLP with risk of CV outcome is stronger and independent of potential confounders in women, compared to men. This female-specificity could arise from a difference in vitamin B6 handling between sexes [46] and may, at least partly, explain the somewhat contradictory data concerning the effects of inflammation on the associations between plasma PLP and CV outcome [5,12]. Moreover, it has been described that vitamin B6 deficiency is able to cause atherosclerosis independent of cholesterol concentrations in female rats, but not in male rats [47]. However, while the sex-specific difference in vitamin B6 handling may be related to ageing, the underlying mechanisms and potential non-CV consequences thereof, remain unclear.

Early observational studies postulating a relationship between vitamin B6 status and risk of CV events have precipitated in numerous randomized controlled intervention studies aiming to assess the potential effects of vitamin B6 supplementation on outcome in various populations and under different circumstances. The majority of these studies did not find a beneficial effect of B-vitamin supplementation on CV outcome [48,49], also not in women [50], albeit some have shown a moderate reduction in stroke risk that was borderline significant at a meta-level [48,49]. However, it is difficult to derive the true effect of vitamin B6 supplementation from these meta-analyses, because of at least the following reasons. First, a combination of B-vitamins, in some cases with omega 3 fatty acids, was usually supplemented. This issue was addressed in the latest network meta-analysis [51], that compared the effects of different B-vitamins and identified the combination of folic acid and vitamin B6 as the potentially most efficacious B-vitamin therapy for the prevention of stroke. Second, most of the intervention studies involved predominantly male participant groups, which likely obscured any female-specific effects. Interestingly, a subgroup meta-analysis of the same studies showed that B-vitamin supplementation significantly reduced the risk of major adverse CV events in study populations with <65% men, but not in those with ≥65% men [52]. However, if B-vitamins would indeed confer CV protection in women, the underlying mechanisms would likely not involve improvement of inflammation or endothelial function [43,53,54]. Of note, intervention studies thus far have mostly been conducted in participants with a history or increased risk of CV disease. From the viewpoint of CV disease prevention, this population may be fundamentally different from a generally healthy one, because it is conceivable that CV damage, once inflicted, cannot or insufficiently be reversed by B-vitamin supplementation [55,56]. 

Strengths of our study include the high number of CV events that enabled us to distinguish between non-fatal and fatal CV events, where previous studies were largely limited to a single outcome. Furthermore, by considering the novel composite marker of low-grade systemic inflammation, GlycA, in addition to the traditional inflammation marker hs-CRP, we were able to more comprehensively assess the role of inflammation in the observed associations. Our study also has several limitations. First, our data are observational in nature. Consequently, they do not allow conclusions on causality of the observed associations. Second, while the confounder-adjusted hazard ratio point estimates point towards a large effect, broad confidence intervals indicate that the precision of the prospective data is limited. Unmeasured factors associated with plasma PLP and CV risk, such as alkaline phosphatase [57], may introduce residual confounding that, when taken into account, could result in borderline hazard ratios becoming non-significant. Third, this study, as with most epidemiologic studies, uses a single baseline measurement for studying the association of variables with outcomes, which in theory could affect the strength and relevance of such associations. However, the intra-class correlation coefficient, an indicator of within-person reproducibility over years, of plasma PLP is excellent, thus allowing for one-exposure assessment of vitamin B-6 status on the long-term [58]. Fourth, it is important to note that our data are limited to CV disease and do not provide information on a possible etiological link between vitamin B6 and non-CV conditions, such as cancer [59,60,61]. However, since inflammation is profoundly interrelated with cancer pathophysiology [62], it seems advisable to include assessment of inflammation in future observational cancer-related studies on vitamin B6.

## 5. Conclusions

In conclusion, we have shown that a low vitamin B6 status, as assessed by plasma PLP concentration, was not independently associated with increased risk of adverse CV outcome in the overall cohort. However, the association between plasma PLP and CV outcome was modified by gender. In women, but not men, this association was independent of potential confounders, including inflammation, underlining the potential cardiovascular importance of striving towards an adequate vitamin B6 status in this subpopulation.

## Figures and Tables

**Table 1:** Characteristics overall and according to vitamin B6 status and their association with plasma pyridoxal 5’-phosphate among 6 249 PREVEND study participants.

Linear Regression Models								
		Vitamin B6 Status According to Plasma PLP Concentration			Univariable		Age- and Sex-Adjusted	
	Total Study Population	Deficient (<20 nmol/L)	Insufficient (20–30 nmol/L)	Sufficient (>30 nmol/L)	Stand. β	*P* for Trend	Stand. β	*P* for Trend
N (% of total study population)	6249 (100.0)	902 (14.4)	1261 (20.2)	4086 (65.4)				
Plasma PLP, nmol/L	37.2 (25.1–57.0)	15.4 (12.6–18.0)	25.0 (22.4–27.3)	49.3 (38.0–71.7)				
**Demographics**								
Age, years	53.0 ± 11.9	56.1 ± 12.3	53.6 ± 12.0	52.2 ± 11.6	0.10	<0.001		
Male gender, n (%)	3018 (48.3)	394 (43.7)	558 (44.3)	2066 (50.6)	0.02	0.21		
BMI, kg/m^2^	26.1 (23.6–29.0)	26.6 (23.9–29.6)	26.3 (23.8–29.4)	25.9 (23.6–28.7)	0.08	<0.001	0.06	<0.001
Vitamin B6 supplementation, n (%)	12 (0.2)	0 (0)	0 (0)	12 (0.2)	0.14	<0.001	0.14	<0.001
Smoking, n (%)								
Never	1857 (29.7)	204 (22.6)	339 (26.9)	1314 (32.2)	*Ref.*		*Ref.*	.
Former	2572 (41.2)	309 (34.4)	496 (39.3)	1767 (43.2)	0.03	0.04	0.02	0.29
Current	1740 (28.2)	377 (42.2)	412 (33.0)	951 (23.6)	0.15	<0.001	0.16	<0.001
Cotinine excretion, µg/24 h	0 (0–493)	10 (0–1399)	0 (0–842)	0 (0–69)	0.20	<0.001	0.22	<0.001
Education, n (%)								
Low	2690 (43.0)	523 (58.0)	593 (47.0)	1574 (38.5)	*Ref.*		*Ref.*	
Middle	1605 (25.7)	199 (22.1)	334 (26.7)	1069 (26.2)	0.08	<0.001	0.07	<0.001
High	1954 (31.3)	180 (20.0)	331 (26.2)	1443 (35.3)	0.16	<0.001	0.14	<0.001
SBP, mmHg	123 (112–136)	127 (114–143)	124 (112–138)	122 (112–135)	0.08	<0.001	0.05	0.002
DBP, mmHg	73 (67–79)	74 (68–80)	73 (67–79)	72 (67–79)	0.05	<0.001	0.03	0.06
Creatinine excretion, mmol/24 h	12.0 (9.9–14.6)	11.5 (9.6–13.9)	11.8(9.8–14.3)	12.1 (10.0–14.9)	0.02	0.15	0.01	0.58
Moderate physical activity, n (%)								
None	942 (15.2)	210 (23.5)	223 (17.9)	509 (12.6)	*Ref.*		*Ref.*	
1 time per week	677 (10.9)	94 (10.5)	141 (11.3)	442 (10.9)	0.07	<0.001	0.06	0.001
>1 time per week	4564 (73.8)	589 (66.0)	885 (70.9)	3090 (76.5)	0.12	<0.001	0.11	<0.001
**Dietary intake**								
Coffee consumer, n (%)	5831 (94.2)	847 (94.6)	1181 (94.6)	3803 (94.0)	0.04	0.004	0.03	0.03
Alcohol consumptions, n (%)								
No/rarely	1541 (24.9)	353 (39.4)	338 (27.1)	850 (21.0)	*Ref.*		*Ref.*	
1–4 per month	1054 (17.0)	181 (20.2)	240 (19.2)	633 (15.7)	0.04	0.02	0.03	0.05
2–7 per week	1973 (31.9)	239 (26.7)	418 (33.5)	1315 (32.5)	0.10	<0.001	0.09	<0.001
2–3 per day	1355 (21.9)	107 (11.9)	217 (17.4)	1031 (25.5)	0.15	<0.001	0.15	<0.001
>3 per day	266 (4.3)	16 (1.8)	35 (2.8)	215 (5.3)	0.10	<0.001	0.10	<0.001
Ethylglucuronide excretion, µg/24 h	144 (0–3751)	15 (0–860)	60 (0–1898)	404 (3–4692)	0.08	<0.001	0.09	<0.001
Potassium excretion, mmol/24 h	68.6 ± 21.9	61.8 ± 21.0	65.60 ± 20.4	70.9 ± 22.1	0.13	<0.001	0.13	<0.001
Urea excretion, mmol/24 h	365 ± 114	341 ± 114	359 ± 107	372 ± 115	0.06	<0.001	0.06	<0.001
**Inflammation**								
Hs-CRP, mg/L	1.3 (0.6–3.0)	2.4 (1.0–5.4)	1.5 (0.8–3.4)	1.1 (0.5–2.5)	0.21	<0.001	0.20	<0.001
GlycA, µmol/L	345 (308–388)	376 (333–426)	355 (317–393)	336 (302–377)	0.22	<0.001	0.21	<0.001
**Glucose homeostasis**								
Diabetes, n (%)	353 (5.7)	79 (8.8)	78 (6.2)	196 (4.8)	0.05	<0.001	0.03	0.02
Glucose, mmol/L	4.8 (4.4–5.3)	4.8 (4.4–5.4)	4.8 (4.4–5.3)	4.8 (4.4–5.3)	0.06	<0.001	0.04	0.01
**Lipids**								
Total cholesterol, mmol/L	5.5 ± 1.0	5.4 ± 1.1	5.4 ± 1.0	5.5 ± 1.0	0.03	0.06	0.05	<0.001
HDL-cholesterol, mmol/L	1.2 (1.0–1.7)	1.2 (1.0–1.4)	1.2 (1.0–1.4)	1.3 (1.1–1.5)	0.05	<0.001	0.05	<0.001
LDL-cholesterol, mmol/L	3.6 ± 0.9	3.6 ± 1.0	3.6 ± 0.9	3.6 ± 0.9	0.01	0.83	0.02	0.18
Triglycerides, mmol/L	1.1 (0.8–1.6)	1.2 (0.9–1.7)	1.1 (0.8–1.6)	1.1 (0.8–1.6)	0.01	0.47	0.01	0.80
total cholesterol:HDL cholesterol ratio	4.6 ± 1.4	4.8 ± 1.4	4.6 ± 1.3	4.5 ± 1.4	0.09	<0.001	0.09	<0.001
**Kidney function**								
Serum creatinine, µmol/L	72.3 ± 18.3	71.1 ± 15.8	71.5 ± 16.1	72.9 ± 19.4	0.03	0.08	0.04	0.01
Cystatin C, mg/L	0.90 ± 0.20	0.96 ± 0.22	0.92 ± 0.20	0.88 ± 0.19	0.13	<0.001	0.12	<0.001
eGFR, mL/min/1,73 m^2^	86.1 ± 17.4	82.7 ± 18.1	85.5 ± 18.1	87.1 ± 16.7	0.08	<0.001	0.05	0.008
Albumin excretion, mg/24 h	8.53 (6.04–15.13)	9.8 (6.3–22.0)	8.7 (6.0–17.3)	8.3 (6.0–13.9)	0.09	<0.001	0.08	<0.001
**Use of drugs, n (%)**								
Antihypertensives	1054 (19.4)	192 (23.6)	255 (23.1)	607 (17.2)	0.05	<0.001	0.02	0.21
Antidiabetics	184 (3.4)	49 (6.0)	39 (3.5)	96 (2.7)	0.05	0.002	0.03	0.03
Statins	339 (6.2)	66 (8.1)	77 (7.0)	196 (5.6)	0.04	0.02	0.02	0.18

Absolute values are presented as mean ± standard deviation, median [interquartile range], or number (percentage). Linear regression models were constructed with log transformed plasma PLP. Abbreviations: stand. β, standardized β; hs-CRP, high-sensitivity C-reactive protein; PLP, pyridoxal 5’-phosphate; ref, reference; BMI, body mass index; SBP, systolic blood pressure; DBP, diastolic blood pressure; HDL, high-density lipoprotein; LDL, low-density lipoprotein; eGFR, estimated glomerular filtration rate.

**Table 2:** Hazard ratios for the associations between plasma pyridoxal 5’-phosphate and vitamin B6 status and cardiovascular outcome, with adjustment for potential confounders.

	Per Increment of Log Transformed Plasma PLP	Vitamin B6 Status According to Plasma PLP Concentration		
		Deficient (<20 nmol/L)	Insufficient (20–30 nmol/L)	Sufficient (>30 nmol/L)
**Composite Cardiovascular Outcome**				
Cases	6205	3868	1163	806
Person-years	48,466	32,068	9636	6762
Events	409	217	97	95
Crude model	0.35 (0.25–0.49)	2.09 (1.64–2.66)	1.49 (1.18–2.66)	1.00 (ref)
Model 1 *	0.53 (0.38–0.74)	1.58 (1.24–2.02)	1.38 (1.09–1.76)	1.00 (ref)
Model 2 ^†^	0.60 (0.42–0.84)	1.44 (1.12–1.85)	1.32 (1.04–1.68)	1.00 (ref)
Model 3 ^§^	0.66 (0.47–0.93)	1.31 (1.02–1.68)	1.25 (0.98–1.59)	1.00 (ref)
**Cardiovascular disease**				
Cases	6205	3868	1163	806
Person-years	48,466	32,068	9636	6762
Events	379	203	92	84
Crude model	0.38 (0.27–0.55)	1.96 (1.52–2.53)	1.51 (1.18–1.93)	1.00 (ref)
Model 1	0.56 (0.40–0.80)	1.51 (1.17–1.96)	1.41 (1.10–1.80)	1.00 (ref)
Model 2	0.63 (0.40–0.91)	1.38 (1.06–1.79)	1.34 (1.04–1.72)	1.00 (ref)
Model 3	0.70 (0.49–1.01)	1.25 (0.96–1.63)	1.27 (0.99–1.63)	1.00 (ref)
**Cardiovascular mortality**				
Cases	6184	4057	1248	902
Person-years	49,911	32,846	9987	7078
Events	77	35	18	24
Crude model	0.15 (0.07–0.32)	3.18 (1.89–5.35)	1.70 (0.96–3.00)	1.00 (ref)
Model 1	0.33 (0.16–0.71)	1.91 (1.14–3.23)	1.41 (0.80–2.49)	1.00 (ref)
Model 2	0.36 (0.17–0.79)	1.77 (1.04–3.02)	1.34 (0.76–2.36)	1.00 (ref)
Model 3	0.39 (0.18–0.85)	1.59 (0.93–2.72)	1.22 (0.69–2.17)	1.00 (ref)

* Adjusted for age and sex; ^†^ as model 1, additionally adjusted for smoking and alcohol consumption; ^§^ as model 2, additionally adjusted for BMI, eGFR, albumin excretion, total cholesterol:HDL-cholesterol ratio, and systolic and diastolic blood pressure. Abbreviations: PLP, pyridoxal 5’-phosphate; ref, reference; BMI, body mass index; HDL, high-density lipoprotein; eGFR, estimated glomerular filtration rate.

**Table 3:** Effect of adjustment for inflammation on the association between plasma pyridoxal 5’-phosphate and cardiovascular outcome.

	Per increment of Log Transformed Plasma PLP	Percentage of Association Explained	Vitamin B6 Status According to Plasma PLP Concentration		
			Deficient (<20 nmol/L)	Insufficient (20–30 nmol/L)	Sufficient (>30 nmol/L)
**Composite cardiovascular outcome**					
Covariate-adjusted *	0.66 (0.47–0.93)		1.31 (1.02–1.68)	1.25 (0.98–1.59)	1.00 (ref)
+ hs-CRP ^†^	0.72 (0.51–1.01)	9	1.23 (0.96–1.59)	1.21 (0.95–1.54)	1.00 (ref)
+ GlycA ^‡^	0.74 (0.53–1.05)	12	1.19 (0.92–1.54)	1.21 (0.95–1.53)	1.00 (ref)
Fully adjusted ^§^	0.75 (0.53–1.07)	14	1.18 (0.91–1.53)	1.20 (0.94–1.53)	1.00 (ref)
**Cardiovascular disease**					
Covariate-adjusted	0.70 (0.49–1.01)		1.25 (0.96–1.63)	1.27 (0.99–1.63)	1.00 (ref)
+ hs-CRP	0.76 (0.53–1.10)	9	1.18 [0.90–1.55)	1.23 (0.96–1.58)	1.00 (ref)
+ GlycA	0.79 (0.55–1.13)	13	1.14 (0.86–1.50)	1.22 (0.95–1.57)	1.00 (ref)
Fully adjusted	0.80 (0.56–1.15)	14	1.13 (0.86–1.49)	1.22 (0.95–1.56)	1.00 (ref)
**Cardiovascular mortality**					
Covariate-adjusted	0.39 (0.18–0.85)		1.59 (0.93–2.72)	1.22 (0.69–2.17)	1.00 (ref)
+ hs-CRP	0.46 (0.21–0.99)	18	1.43 (0.83–2.48)	1.17 (0.66–2.08)	1.00 (ref)
+ GlycA	0.47 (0.21–1.02)	21	1.39 (0.80–2.43)	1.19 (0.67–2.12)	1.00 (ref)
Fully adjusted	0.48 (0.22–1.05)	23	1.37 (0.78–2.39)	1.17 (0.66–2.08)	1.00 (ref)

* Adjusted for age, sex, smoking, alcohol consumption, BMI, eGFR, albumin excretion, total cholesterol:HDL-cholesterol ratio, and systolic and diastolic blood pressure; ^†^ as the covariate-adjusted model, additionally adjusted for hs-CRP; ^‡^ as the covariate-adjusted model, additionally adjusted for GlycA; ^§^ as the covariate-adjusted model, additionally adjusted for both hs-CRP and GlycA. Abbreviations: hs-CRP, high-sensitivity C-reactive protein; PLP, pyridoxal 5’-phosphate; ref; reference; BMI, body mass index; HDL, high-density lipoprotein; eGFR, estimated glomerular filtration rate.
